# Data Collection for Dilute Protein Solutions via a Neutron Backscattering Spectrometer

**DOI:** 10.3390/life12050675

**Published:** 2022-05-02

**Authors:** Taiki Tominaga, Hiroshi Nakagawa, Masae Sahara, Takashi Oda, Rintaro Inoue, Masaaki Sugiyama

**Affiliations:** 1 Neutron Science and Technology Center, Comprehensive Research Organization for Science and Society (CROSS), 162-1 Shirakata, Ibaraki 319-1106, Japan; m_sahara@cross.or.jp; 2 Materials Sciences Research Center, Japan Atomic Energy Agency, Ibaraki 319-1195, Japan; nakagawa.hiroshi@jaea.go.jp; 3 J-PARC Center, Japan Atomic Energy Agency, Ibaraki 319-1195, Japan; 4 Department of Life Science, Rikkyo University, Nishi-Ikebukuro, Toshima-ku, Tokyo 171-8501, Japan; 5065494@rikkyo.ac.jp; 5 Institute for Integrated Radiation and Nuclear Science, Kyoto University, Kumatori, Sennan-gun, Osaka 590-0494, Japan; rintaro@rri.kyoto-u.ac.jp (R.I.); sugiyama.masaaki.5n@kyoto-u.ac.jp (M.S.)

**Keywords:** protein solution, dynamics, QENS, back scattering spectrometer, sample cell, boehmite

## Abstract

Understanding protein functions requires not only static but also dynamic structural information. Incoherent quasi-elastic neutron scattering (QENS), which utilizes the highly incoherent scattering ability of hydrogen, is a powerful technique for revealing the dynamics of proteins in deuterium oxide (D_2_O) buffer solutions. The background scattering of sample cells suitable for aqueous protein solution samples, conducted with a neutron backscattering spectrometer, was evaluated. It was found that the scattering intensity of an aluminum sample cell coated with boehmite using D_2_O was lower than that of a sample cell coated with regular water (H_2_O). The D_2_O-Boehmite coated cell was used for the QENS measurement of a 0.8 wt.% aqueous solution of an intrinsically disordered protein in an intrinsically disordered region of a helicase-associated endonuclease for a fork-structured type of DNA. The cell was inert against aqueous samples at 283–363 K. In addition, meticulous attention to cells with small individual weight differences and the positional reproducibility of the sample cell relative to the spectrometer neutron beam position enabled the accurate subtraction of the scattering profiles of the D_2_O buffer and the sample container. Consequently, high-quality information on protein dynamics could be extracted from dilute protein solutions.

## 1. Introduction

Many proteins possess a hierarchical structure, and their static hierarchical structures are responsible for the efficient expression of functions, such as substrate reactions [1,2,3,4,5,6,7]. In recent years, an increasing number of studies have focused on their dynamics to explore the origin of this functional expression [8,9]. In terms of dynamical hierarchies, the discussion encompasses the rotation and vibration of Å-scale atoms, nanometer-scale rotation and translation of functional groups or amino acid units, intra-motif dynamics formed by the secondary structure of proteins, and thermal fluctuation oscillation, rotation, and translation between domains.

The time scale of dynamics is of the order of picoseconds and microseconds for the vibrational motion of atoms and the expression of functions, respectively. Thus, a very wide range of time scales should be covered by experimental data. Mössbauer spectroscopy (10^−10^–10^−7^ s) [10] and nuclear magnetic resonances (10^−11^–10^−3^ s) [11] provide dynamic information, while the complementary use of neutron scattering methods such as neutron spin echo, neutron back scattering (NBS), and time of flight coupled with deuterium labeling can simultaneously provide unique information about dynamics on spatial scales from Å to tens of nanometers, and on the order of picoseconds to hundreds of nanoseconds [8,9].

Information concerning the dynamics of dry proteins is only available for local motions, and powdered hydrated proteins with a tiny fraction of water adsorbed provide greater dynamic information than dry forms [12,13,14,15,16]. However, they do not provide information reflecting the whole picture compared to systems with large motions, such as domain motions in protein solutions [9]. In an in vivo environment, numerous proteins are crowded with several substances and are in an undiluted state. It is essential to understand the dynamics of proteins under these conditions. D_eff_(*Q*), which is directly experimentally evaluated via QENS, is expressed as D_eff_(*Q*) = D_0_(*Q*) H(*Q*)/S’(*Q*) [17], where D_0_(*Q*) is the diffusion constant at the infinite dilution that we need to discuss, S’(*Q*) is the inter-particle interference, and H(*Q*) is the hydrodynamic function. Under dilute conditions, these terms are negligible (i.e., H(*Q*)~1, S’(*Q*)~1) and *D*_0_(*Q*) can be considered equivalent to D_eff_(*Q*). Although it is essential to understand the dynamics of proteins under these conditions, information regarding the dynamics of pure proteins, excluding interference terms such as particle interference and fluid interactions, can only be discussed under dilute concentrations. To obtain statistically acceptable scattering signals from dilute protein solution samples, it is necessary to (i) utilize instruments with high signal-to-noise ratios (S/N), (ii) minimize uncertainties that could influence the stability of samples (e.g., interactions with sample cells), and (iii) correctly subtract non-essential scatterings such as empty cells and buffers.

Neutron back scattering, which is categorized as a quasi-elastic neutron scattering (QENS) technique, mainly provides incoherent scattering information on a spatiotemporal scale from a few Å to a few nm and from 10 ps to sub-nanoseconds. Owing to its energy range, high resolution, high intensity, and high S/N, the state-of-the-art NBS instrument is suitable for studying the dynamics of proteins. Using a time-of-flight, near-backscattering spectrometer (BL02), at the Materials and Life Science Experimental Facility at J-PARC, Japan [18], Fujiwara and Matsuo et al. have published studies [19,20,21,22,23,24] on protein solutions, including an experiment with the world’s lowest concentration of α-Synuclein at that time (9.5 mg/mL (~0.95 wt.%)) [20]. In recent years, Inoue et al. [25] and Nakagawa et al. [26] reported studies on protein dynamics within protein solutions.

In this study, for appropriate QENS data collection, we carefully acquired data and validated the temperature variation of protein dynamics in dilute protein solutions, focusing on the points of selection of the sample container and reproducibility of the sample container position against the beam position of the spectrometer. Aluminum is conventionally used as a sample cell material for QENS. For aqueous samples, chemical reactions between aluminum and water should be considered. The chemical reactions between the aluminum cell and the aqueous sample were previously summarized, demonstrating that the reaction with aluminum can not only corrode the sample cell, but also change the sample conditions (water concentration and/or ionic environment, and/or pH) [27]. It should be noted that these changes could affect the stability of the sample, thereby deteriorating the quality of the resulting scattering profile. To overcome this problem, we adopted a boehmite coating of aluminum cells, which is expected to be corrosion-resistant over a wide temperature range [27,28]. To further suppress cell-derived incoherent scattering, a boehmite coating with deuterium oxide (D_2_O) was used in this study. To extract and analyze weak protein scattering profiles from dilute protein solutions, reliable scattering profiles of protein solution, its buffer solution, sample cell, and resolution function are required. Owing to the difficulty in using the same container during the allotted measurement time (beam time), the utilization of the same equivalent container and installation at the same sample location is essential for obtaining pure protein scattering profiles from dilute solutions. Through our developed QENS measurement procedures, we present QENS profiles from dilute solutions of intrinsically disordered proteins at several temperatures.

## 2. Materials and Methods

### 2.1. Sample Preparation of Hef-IDR Solutions

The intrinsically disordered region of *Thermococcus kodakarensis* Hef (Hef-IDR, residues 493–593) was expressed in *Escherichia coli* cells and purified. Details are provided in the literature [25]. The Hef-IDR was dialyzed into a D_2_O buffer solution (pD = 7.1) consisting of 10 mM HEPES, 100 mM NaCl, and 0.1 mM EDTA prior to QENS measurement, and the concentration was adjusted to 0.8 wt.% using the D_2_O buffer.

### 2.2. D_2_O-Boehmite Coated Sample Cells

The sample container comprised outer and inner cylinders made of aluminum (Al 1070) [27]. The inner diameter of the outer cylinder was 14 mm, the outer diameter of the inner cylinder was 13 mm, and the wall thicknesses of both the inner and outer cylinders were 0.25 mm. The arithmetic mean roughness, *R*_A_, of each cylinder was approximately 0.28 µm (which is the same as that of the regular machining surface in the literature [27]). By loading the liquid sample into the gap between these cylinders (the space created by the inside of the outer cylinder and the outside of the inner cylinder), the sample was formed into an annular shape. The differences in wall thickness between multiple sample cells used for the protein solution, buffer, and empty cells should be small. Hence, as a simple method, we selected outer and inner cylinders with similar weights (weight tolerance: <0.3%) and used them for the QENS experiments.

After removing contaminants from the surfaces of the aluminum cells in contact with the samples (inside of the outer cylinder and outside of the inner cylinder) with ethanol-impregnated Kimwipes (Kimberly-Clark, Irving, TX, USA), each cell surface was boehmite coated with D_2_O (DLM-4-99.8-1000 (D, 99.8%), Cambridge Isotope Laboratories, Inc., MA, Tewksbury, USA) at temperatures greater than 368 K using dry blocks (D1200, Labnet International, Inc., Edison, NJ, USA). To minimize the evaporation of D_2_O and the temperature decrease during the boehmite treatment, D_2_O was supplied gradually from a container with a slightly loose screw cap. The boiling time was 15 h to reach a near-equilibrium change in film thickness over time, based on literature [29]. The surfaces of the boehmite-coated cells were similarly cleaned with ethanol-impregnated Kimwipes before the experiment.

The effect of the boehmite coating on the QENS profiles was evaluated using BL02 [18]. A proton beam power of 600 kW was incident on the neutron target. The wavelength of the incident neutrons was 6.32 ± 2.07 Å. The energy resolution was 3.6 µeV using Si 111 analyzers. The covered ranges of the scattering vector *Q* (in Å^−1^) and energy transfer *E* (in meV) were 0.13 < *Q* < 1.7 and −0.01 < *E* < 0.02, respectively. The exposure times were approximately 3.9 h. The acquisition and analysis of data were the same as in a previous paper [27]. The data were obtained after subtracting the background profile of the instrument and correcting for detector efficiency using a vanadium standard.

### 2.3. QENS Measurements for Protein Solutions

QENS measurements for the protein solutions were performed using BL02 [18]. This instrument has an excellent S/N > 10^5^ and is suitable for collecting the scattering profiles of solutes in dilute protein solutions. A proton beam power of 600 kW was incident on the neutron target. The wavelength of the incident neutrons was 6.32 ± 2.07 Å. The energy resolution was 12 µeV using Si 111 analyzers. The covered *Q* and *E* values were 0.13 < *Q* < 1.7 and −0.4 < *E* < 1, respectively.

Depending on the neutron beam size (15 × 40 mm), a 0.8 mL sample solution was loaded into the gap (0.5 mm) between the outer and inner cylinders of the boehmite-coated aluminum cell described in Section 2.2 and sealed with a metal O-ring. Sample cells containing the Hef-IDR solution and its buffer solution were mounted on the bottom end of a center stick, and the stick was installed into the BL02 standard top-loading cryostat. The sample cells were subjected to QENS measurements at 283, 298, 323, 343, and 363 K for the protein solution and buffer. The scattering profiles of the empty cell and resolution were collected at 283 K. The exposure time for each measurement was ~7 h.

A set of boron nitride (neutron absorber) masks was attached to the sample cell to collect only appropriate scattering data. The center stick was eccentric within 2 mm at the sample position. Therefore, the center stick was carefully placed in the cryostat to ensure that the orientation of the center stick was always the same, ensuring that the neutron path lengths of the protein solution, buffer, empty cell, and vanadium standard required for analysis were the same.

The *Q*-*E* maps of the Hef-IDR solution including the cell, buffer with the cell, empty cell, and vanadium were obtained by subtracting instrumental background profiles and correcting for detector efficiency using a vanadium standard. The dynamic structure factor, *S*(*Q*, *E*), of the samples was corrected for the detector efficiency using a vanadium standard. The dynamic structure factor of proteins in the protein solution was extracted from the *Q*-*E* maps using Equation (1).

*S*_p_(*Q*, *E*) = {*S*_p+b+c_(*Q*, *E*) − A × *S*_c_(*Q*, *E*)} − B × {*S*_b+c_(*Q*, *E*) − C × *S*_c_(*Q*, *E*}, (1)

where *S*_p_(*Q*, *E*), *S*_p+b+c_(*Q*, *E*), *S*_b+c_(*Q*, *E*), and *S*_c_(*Q*, *E*) are the dynamic scattering factors of the protein, protein + buffer + cell, buffer + cell, and cell, respectively, and A, B, and C are their coefficients. The scattering profile of the empty cell after absorption correction was subtracted to account for individual empty cell differences, and subsequently, the D_2_O buffer was subtracted from those of the protein solutions based on their volume fractions to obtain the protein dynamics. A and C incorporate the absorption coefficient of the cell and the adjustment constant for individual empty cell differences, and B incorporates the absorption coefficient of the buffer in the protein solution, its volume fraction, specific volume, etc. When the absorption coefficient of the cell is small and the solute concentration is low, all coefficients are approximately equal to 1.

## 3. Results and Discussions

### 3.1. Scattering Profile of D_2_O-Boehmite Coating Cell

**Figure 1:**
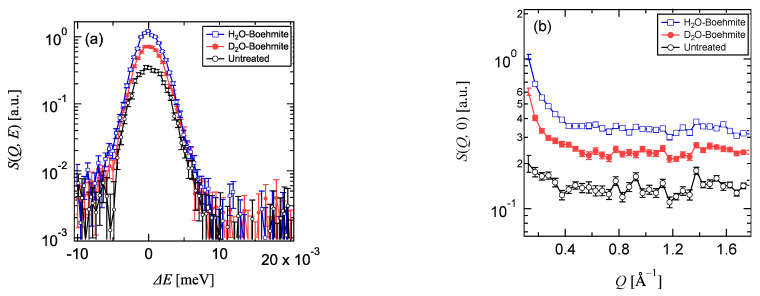
(**a**) *S*(*Q*, *E*) of H_2_O-Boehmite coated, D_2_O-Boehmite coated, and untreated Al cells at -0.01 < Δ*E* < 0.02, plotted on average at 0.13 < *Q* < 1.7. (**b**) *S*(*Q*, 0) at 0.13 < *Q* < 1.7.

Figure 1a shows *S*(*Q*, *E*) of the D_2_O-Boehmite coated Al cell at −0.01 < Δ*E* meV < 0.02, plotted on average at 0.13 < *Q* Å^−1^ < 1.7 after subtracting the background profile of the instrument and correcting for detector efficiency using a vanadium standard, and the profiles of an H_2_O-Boehmite coated cell and an untreated cell were also appended to the graph as references. Owing to the fact that the boiling times for the boehmite treatment with H_2_O [27,28] and D_2_O are 1 h and 15 h, respectively, the thickness of the formed boehmite film based on the literature values can be estimated to be roughly 0.5 µm and 6.5 µm [29].

The elastic intensity of the boehmite-coated cell by D_2_O was approximately 60% of that of H_2_O. Considering the incoherent cross-section of hydrogen and deuterium (H: 82.02 barn, D: 2.05 barn) and the estimated thickness of the D_2_O-Boehmite, this result is reasonable; furthermore, it was found that the H contamination in the D_2_O-Boehmite was small. The boehmite coatings can be expected to provide corrosion resistance even in the case of thin films [28,29]. However, in QENS experiments that require measurements over long periods of time, especially under high temperatures, the boehmite may form using water in the aqueous solution samples. Such high-temperature experiments should be conducted using cells with a sufficiently thick layer of boehmite, obtained by boiling for 15 h or more. While the scattering intensity of the D_2_O-Boehmite-coated cell is higher than that of the untreated aluminum cell, the chemical reaction between water and aluminum under high temperatures can be suppressed [28,29], and the *Q*-dependence is small for *Q* > 0.3 (Figure 1b), making it useful as an empty cell for aqueous solution samples over a wide temperature range.

### 3.2. Scattering Profiles of the Hef-IDR Solution, the Buffer, and the Empty Cell

**Figure 2:**
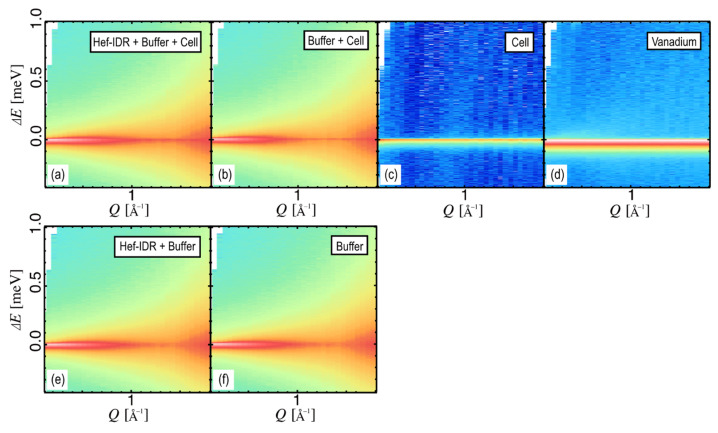
*Q*-*E* profiles of (**a**) Hef-IDR + Buffer + Cell, (**b**) Buffer + Cell, (**c**) Cell, and (**d**) Vanadium, at −0.4 < Δ*E* < 1 and at 0.13 < *Q* < 1.7. Both (**e**) Hef-IDR + Buffer and (**f**) Buffer are obtained by subtracting (**c**) from (**a**,**b**). The intensity shown in the color chart indicates that red is large and blue is small.

The *Q*-*E* maps of the Hef-IDR solution with the cell, its buffer with the cell, empty cell, and vanadium (resolution) at 283 K are shown in Figure 2a–d, respectively. The *x*-, *y*-, and *z*-axes represent the scattering vector *Q*, energy transfer, and scattering intensity, respectively; the intensity is larger in red and smaller in blue. The empty cell and vanadium had a large elastic component, and the scattering in the inelastic region was small. In the buffer solution, D_2_O was mobile, and in the region where *Q* > 1.5 Å^−1^, the profile derived from the first correlation peak of D_2_O could be observed. The profile in Figure 2a is slightly yet clearly different from that shown in Figure 2b, indicating that Hef-IDR-derived QENS signals were experimentally detected.

The scattering profiles of empty cells mainly correspond to elastic scattering (Figure 2c). Therefore, it is reasonable to assume that the elastic scattering (Δ*E*~0) of the buffer solution in Figure 2b for *Q* > 1.5 is derived from the empty cells. We subtracted the scattering profile of the empty cell, as shown in Figure 2c, from the profile in Figure 2b, using a C coefficient of 0.93 in Equation (1) from all *Q* ranges (Figure 2f). Considering the absorption correction of the empty cell by numerical calculations [30], we subtracted the scattering profile of the empty cell, as shown in Figure 2c, from those in Figure 2a,b, and obtained Figure 2e,f. Considering the absorption coefficient and incoherent scattering ability of the main constituent of the cell, Al, as well as its scattering volume, its contribution to the scattering profile is small [30]. Therefore, it is reasonable to assume that the scattering intensity in Figure 2c is due to the incoherent scattering of elements (H and D) in the boehmite on the Al surface [26]. Due to the possible influence of the surface morphology of the Al, it is difficult to precisely estimate these incoherent scattering components through numerical calculations; however, if the cells are approximately identical and the sample includes a sufficiently dilute solution, the same coefficient can be used (i.e., A = C in Equation (1)). The profile shown in Figure 2c was subtracted from that shown in Figure 2a in the same manner (Figure 2e).

### 3.3. Temperature Dependence of Protein Dynamics in the Hef-IDR Protein Solutions

**Figure 3:**
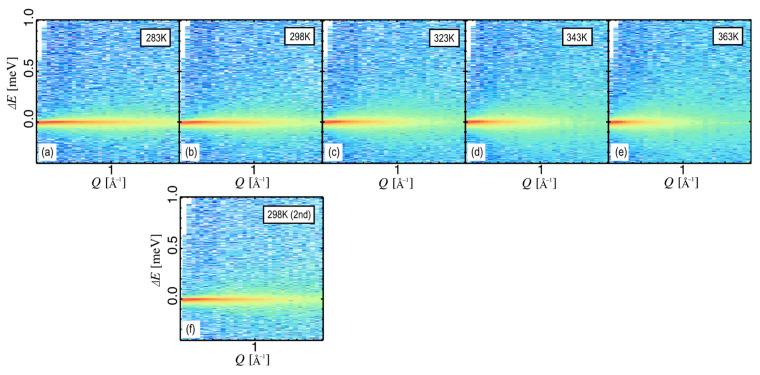
*Q*-*E* temperature dependence of the Hef-IDR within the Hef-IDR solution at −0.4 < Δ*E* < 1 and at 0.13 < *Q* < 1.7. The temperatures are (**a**): 283 K, (**b**): 298 K, (**c**): 323 K, (**d**): 343 K, (**e**): 363 K, and (**f**): 298 K for the second time, respectively. The intensity shown in the color chart indicates that red is large and blue is small.

In the buffer subtraction procedure, we applied an excluded volume fraction that considered the protein concentration, which in the case of our dilute protein solution was treated as approximately one [31]. As Hef-IDR is a highly mobile protein with a small molecular weight, its mobility is outside of the energy window (−0.4 < Δ*E* < 1) at higher *Q* and at higher temperatures [25]. Thus, the buffer profiles were subtracted so that the *Q*-*E* profiles were flat at high *Q*; we subtracted the scattering profiles of the buffer from those of the protein and buffer. The coefficient of the buffer (coefficient B in Equation (1)) was 0.95 ± 0.03 throughout the tested temperature range, which is a reasonable value considering the protein concentration without temperature dependence. As the temperature increased, the QENS profile broadened, indicating that the proteins became more mobile with increasing temperature (Figure 3a–e). The 363 K experiment was followed by a repeat of the QENS experiment at 298 K to evaluate the effect of the high-temperature experiments on the sample in the D_2_O-Boehmite coated aluminum cell. The results showed that the profiles were in good agreement and the effect was small (Figure 3b,f). Thus, we succeeded in obtaining very reliable data on the temperature dependence of the dynamics of Hef-IDR in extremely dilute 0.8 wt.% Hef-IDR solutions at 283–363 K.

**Figure 4:**
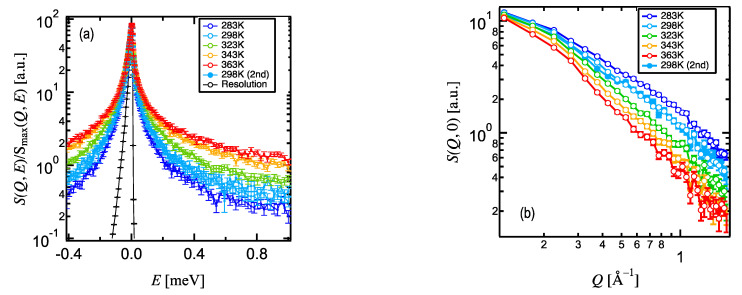
(**a**) *S*(*Q*, *E*) temperature dependence of the Hef-IDR within the Hef-IDR solution, plotted on average at 0.13 < *Q* < 1.7. (**b**) *S*(*Q*, 0) at 0.13 < *Q* < 1.7.

The *S*(*Q*, *E*)/*S*_max_(*Q*, *E*) averaged over 0.13 < *Q* < 1.7 of Hef-IDR in Hef-IDR solution broadened with the increase in temperature (Figure 4a), and the *Q* dependence of the intensity at *E*~0, *S*(*Q*, 0) also increased with the increase in temperature (Figure 4b). Each of the 298 K profiles in Figure 4 overlaps well with each other. This result clearly revealed that the D_2_O-Boehmite-coated cells can extract the required protein dynamics while suppressing chemical reactions such as hydroxylation between the aqueous sample and the sample cells at high temperatures. This indicates that the D_2_O-Boehmite coated cells are advantageous for aqueous samples (Figure 3b,f).

The equality of coefficients A and C in Equation (1) is ensured by the identity of the sample cells and the positional reproducibility of the installed cells. In this study, the identity of the sample cell is ensured by the weight of the cell and the identity of the boehmite processing time; however, it would be better if this identity could be evaluated based on the same absorption coefficient of the sample cell, as derived from neutron transmission experiments. The coefficients A and C showed scattered values (0.85 ± 0.11) through the tested temperature range. This discrepancy cannot be explained by a change in the sample position, which is assumed by the linear expansion coefficient of the metal of the stick. We suppose that the eccentricity of the center stick causes the temperature dependence of the sample position to deviate. This issue can be addressed by obtaining data on the temperature dependence of empty cells as well as protein solutions and buffers and by improving the positional reproducibility of the sample cells for temperature variation experiments. 

## 4. Conclusions

The data on proteins in dilute protein solutions can be collected by using sample cells with no or little interaction between the sample and the cell by selecting cells with small individual differences, and by paying close attention to the reproducibility of the sample position. In particular, the aluminum cell coated with D_2_O boehmite proved to be very advantageous as a sample container for QENS experiments in aqueous solution systems over a wide temperature range. With careful experiments, the dynamics of Hef-IDR were successfully obtained in a 0.8 wt.% Hef-IDR solution in the range of 283–363 K. It was also confirmed that no chemical reaction occurred between the boehmite-coated aluminum container and the sample at the high temperature of 363 K, which did not affect the scattering profile. Although the reproducibility of the sample cell position under temperature change experiments was not fully guaranteed, the installation of guides on the top and bottom of the sample cell (PEACE [32]), as a sample changer, can improve the position dependency of the sample cells. 

In the case of QENS experiments using partially deuterated proteins, an experimental system with more precise sample position reproducibility in a wide temperature range is required because the scattering intensity of the portion of interest decreases.

## Data Availability

The datasets generated and analyzed during the current study are available from the corresponding authors on reasonable request.
